# Transfer of magnetic anisotropy in epitaxial Co/NiO/Fe trilayers

**DOI:** 10.1038/s41598-024-51896-w

**Published:** 2024-01-19

**Authors:** M. Szpytma, M. Ślęzak, W. Janus, H. Nayyef, T. Ślęzak, A. Mandziak, M. Zając, D. Wilgocka-Ślęzak, T. O. Menteş, M. Jugovac, A. Locatelli, A. Kozioł-Rachwał

**Affiliations:** 1 Faculty of Physics and Applied Computer Science, AGH University of Krakow, Krakow, Poland; 2 National Synchrotron Radiation Centre SOLARIS, Jagiellonian University, Krakow, Poland; 3 Jerzy Haber Institute of Catalysis and Surface Chemistry, Polish Academy of Sciences, Krakow, Poland; 4 Elettra – Sincrotrone Trieste, Basovizza, Trieste, Italy

**Keywords:** Surfaces, interfaces and thin films, Magnetic properties and materials

## Abstract

The magnetic properties of Co(10 Å)/NiO(40 Å)/Fe trilayer epitaxially grown on W(110) substrate were investigated with use of x-ray magnetic linear dichroism (XMLD) and x-ray magnetic circular dichroism (XMCD). We showed that magnetic anisotropy of Fe film that can be controlled by a thickness-driven spin reorientation transition is transferred via interfacial exchange coupling not only to NiO layer but further to ferromagnetic Co overlayer as well. Similarly, a temperature driven spin reorientation of Fe sublayer induces a reorientation of NiO spin orientation and simultaneous switching of the Co magnetization direction. Finally, by element specific XMCD and XMLD magnetic hysteresis loop measurements we proved that external magnetic field driven reorientation of Fe and Co magnetizations as well as NiO Néel vector are strictly correlated and magnetic anisotropy fields of Fe and Co sublayers are identical despite the different crystal structures.

## Introduction

Magnetic anisotropy (MA) is a feature of magnetic materials that plays a crucial role in technological applications. In thin films MA can be determined by magnetocrystalline, dipolar or magneto-elastic effects^1^. Additional contributions to MA appear for thin film heterostructures via so called magnetic proximity effects for which the presence of neighboring magnetic or/and non-magnetic layers can induce interface anisotropy^2^, interlayer exchange coupling^3–5^ or exchange anisotropy^6^. The latter describes the interaction between two magnetically ordered layers, in particular the coupling at the interface between ferromagnet (FM) and antiferromagnet (AFM) that has been widely utilized in spin valves^7^ and magnetic tunnel junctions^8^. Exchange coupling at AFM/FM interface can be used to manipulate the magnetic properties of both FM and AFM layers. For fully compensated (001) surface of NiO, an exchange interaction at the Fe/NiO interface caused the AFM domain structure of NiO to follow the FM domains of Fe^9^. Furthermore, for Py/IrMn and CoO/Fe it was shown that a nonuniform magnetization state in the FM layer can modify the spin structure of AFM^10,11^.

Among the most intensively studied AFM/FM interfaces are those which contain an antiferromagnetic NiO layer. Bulk NiO crystallizes in a cubic NaCl structure. Below its Néel temperature (T_N_) of 523 K, the magnetic moments of Ni^2+^ ions align ferromagnetically within the (111) planes, while the adjacent (111) planes are coupled antiferromagnetically. Recent demonstration of long-distance spin transport^12^, spin Hall magnetoresistance^13,14^ and current-induced switching in NiO/Pt^15,16^ revealed the potential of NiO as active element in spintronic devices. Experimental research on the FM/NiO structure does not unambiguously conclude about the relative orientation of magnetic moments of FM and AFM layers. Although, a perpendicular orientation of the FM and AFM easy axes was predicted for the ideal FM/AFM interface^17^, both collinear and non-collinear coupling have been reported for FM/NiO with [001] orientation of NiO so far^18–21^. A collinear coupling at the FM/AFM interface is often associated with an exchange bias effect which manifests itself as a horizontal shift of magnetic hysteresis loop and enhanced coercivity of the FM layer^22^. More complicated magnetic structures can be formed in FM/AFM/FM trilayers in which for metallic AFM spacer, the combination of exchange bias coupling with a long-range Ruderman–Kittel–Kasuya–Yosida (RKKY) coupling can exist^3,23–27^. For comparable magnitudes of exchange bias and interlayer exchange coupling, a complex magnetization switching process was demonstrated in Fe/Mn/Co^23^. In trilayers with insulating AFM (iAFM) interlayer, the interaction between FM layers due to the conduction electrons of the spacer is excluded. Interlayer exchange coupling which arises from the spin-dependent electron-tunneling process causes monotonic decay of the indirect coupling strength together with an increase in the thickness of insulating spacer in the regime of ultrathin spacer layers^5,28^. Simultaneously, in FM/iAFM/FM, magnetostatic interactions, the spin structure of the iAFM layer, as well as interfacial coupling between FM and iAFM should be considered^29–33^.

In this work we focused on the magnetic properties of Co/NiO/Fe trilayer epitaxially grown on W(110) substrate. As we showed previously in NiO/Fe/W(110)^34,35^, MA and orientation of NiO spins within (111) plane can be tuned by magnetic properties of the underlying Fe layer. In particular, the direction of Fe spontaneous magnetization that can be controlled by a thickness and temperature driven spin reorientation transition was directly imprinted into the NiO spin structure. Here we proved that the easy axis direction and strength of anisotropy field in the Fe layer is transferred through NiO to a distant Co film which reveals an existence of considerable exchange coupling at both NiO/Fe and Co/NiO interfaces. As a consequence, modulation of the magnetic state and anisotropy of Co layer can be triggered by variation of Fe thickness or temperature.

## Experimental

**Figure 1:**
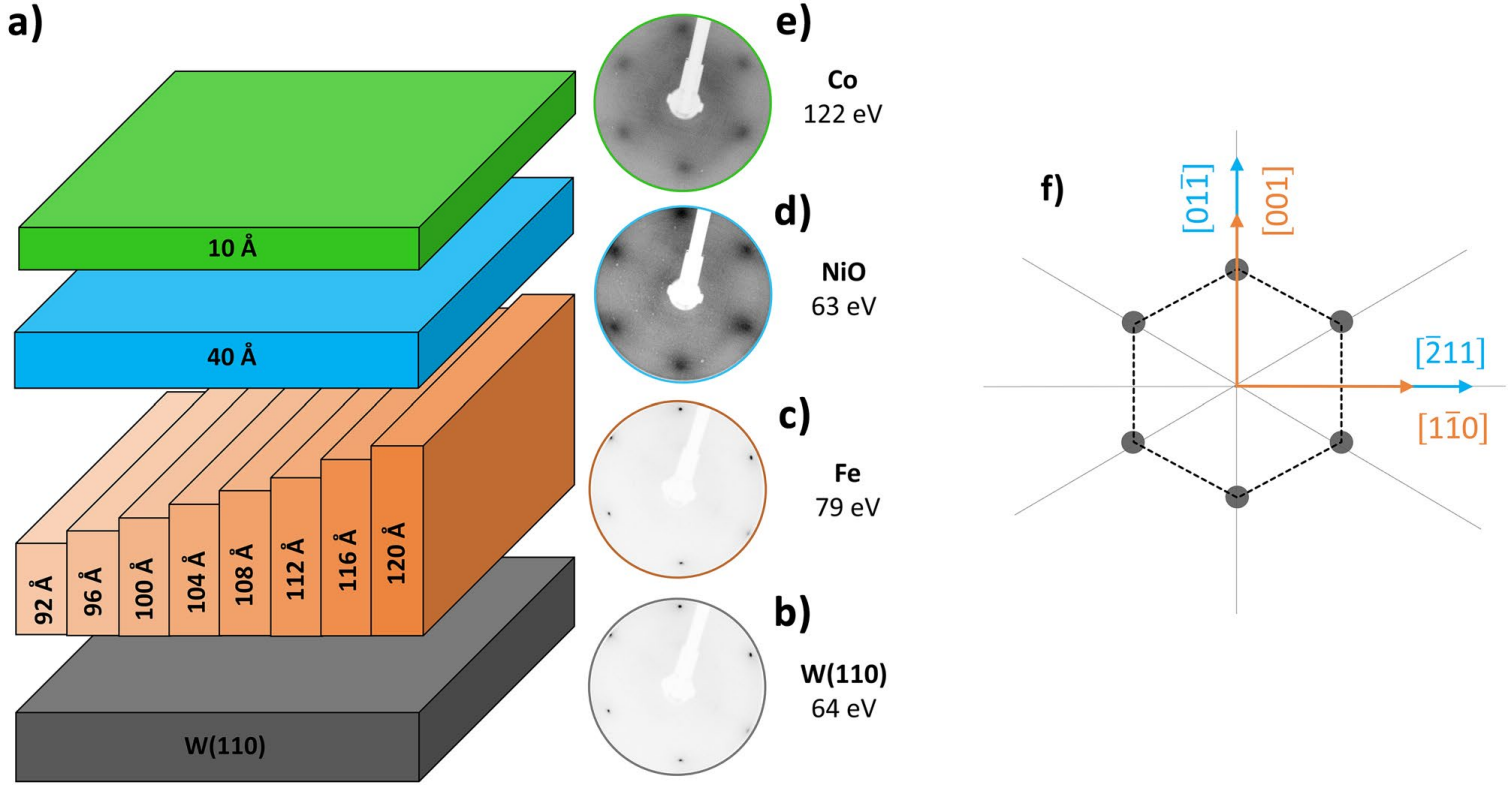
(**a**): Schematic drawing of the sample, (**b**–**e**), from the bottom: LEED patterns collected on W(110) substrate (**b**), after the deposition of Fe (**c**), NiO (**d**) and Co (**e**). For each pattern an electron energy at which image was collected is shown; (**f**) schema of hexagonal diffraction pattern of NiO and Co (grey dots). Relative orientation of Fe and NiO in-plane directions within Fe(110) and NiO(111) planes were marked by brown and blue arrows, respectively.

The samples were prepared in the ultra-high vacuum (UHV) chamber equipped with molecular beam epitaxy (MBE) evaporators. A bcc W(110) single crystal was used as a substrate. A standard cleaning procedure was performed to remove carbon impurities from the W surface^36^. Cleanliness of the W substrate surface was confirmed by low energy electron diffraction (LEED) (Fig. 1b). The Fe(110) layers were grown by MBE on W(110) at room temperature and annealed at 675 K for 15 min. A motorized shutter was moved in front of the sample throughout the Fe films growth. As a result, distinct 1 mm wide strips with varying Fe thicknesses (*d*_*Fe*_) ranging from 92 Å to 120 Å were formed on the sample. In studied Fe thickness range, the spin reorientation transition (SRT) from [$1\overline{1}0$] to [001] occurs as a function of increasing Fe thickness^37,38^ or decreasing temperature. LEED pattern of Fe after annealing confirmed the existence of unreconstructed bcc Fe(110) surface (Fig. 1c). The same LEED pattern was visible on all of the regions with different Fe thicknesses. The 40 Å thick NiO layer was grown on top of Fe via reactive deposition of Ni in a molecular oxygen atmosphere under a partial pressure of 1 × 10^–6^ mbar and a substrate temperature of 300 K. LEED pattern collected after the deposition of NiO shows six-fold symmetry, confirming that NiO(111) surface structure was formed regardless of the Fe underlayer thickness (Fig. 1d). Similarly to previously reported results ^35^ we obtained the following relation between crystallographic in-plane directions of Fe and NiO: Fe[001]‖NiO[${01}\overline{1}$], Fe[${1}\overline{1}{0}$]‖NiO[$\overline{2}{11}$] (Fig. 1f). Growth of 10 Å thick Co layer at room temperature followed the NiO deposition. Figure 1a shows the schematic drawing of the sample. The LEED pattern collected on the Co surface shows six-fold symmetry expected from the (0001) surface orientation of hexagonal Co (Fig. 1e). To compare the magnetic properties of NiO with a thickness of 40 Å and 8 Å a similar sample, but with 8 Å thick NiO layer was prepared, following the same methodology.

Magnetic properties of Co/NiO/Fe trilayer were characterized by x-ray magnetic circular dichroism (XMCD) and x-ray magnetic linear dichroism (XMLD)^20,39^. X ray absorption (XA) spectra were collected at the PIRX beamline^40^ of the National Synchrotron Radiation Center SOLARIS^41^. The XA spectra were measured in the total-electron-yield (TEY) detection mode by measuring the sample drain current. As TEY mode is surface sensitive, we probe only few top-most nm of Fe in XAS measurements. To probe the magnetic properties of NiO, XA spectra were collected using a linearly polarized x-ray beam with a photon energy corresponding to the Ni L_2_ edge^42^. The measurements were performed in normal and grazing incidence geometry of the x-rays, with the linear polarization direction in the sample plane. The spin orientation of the Fe and Co sublayers was studied by collecting the XA spectra for the photon energy tuned to the Fe and Co L_2,3_ absorption edges using two circular polarizations with opposite helicity. To ensure sampling from a region of uniform Fe thickness, the incident radiation spot size was restricted to 200 µm for all of the XAS measurements. Visualization of the magnetic domain structure in the FM and AFM layers was performed using x-ray photoemission electron microscopy (XPEEM). Prior to the XPEEM measurements the sample was capped with 1 nm of Au to prevent oxidation of Co layer. We proved that the capping layer does not influence magnetic properties of the stack (Fig. S4, Supplemental Materials). XPEEM images were collected at the CIRCE beamline of the ALBA Synchrotron Light Facility^43^ and the Nanospectroscopy beamline of the Elettra synchrotron^44^. In both setups, the x-rays were incident on the sample at a grazing angle of 16° from the surface plane. The differential XMCD-PEEM images of Fe and Co layers were obtained at the respective L_3_ absorption edges by taking the difference between images collected with the two opposite circular polarizations. The NiO XMLD-PEEM image was obtained by calculating the asymmetry from images taken at two absorption energies corresponding to the peaks within the Ni L_2_ edge. (see Supplemental Materials for details).

## Results and discussion

**Figure 2:**
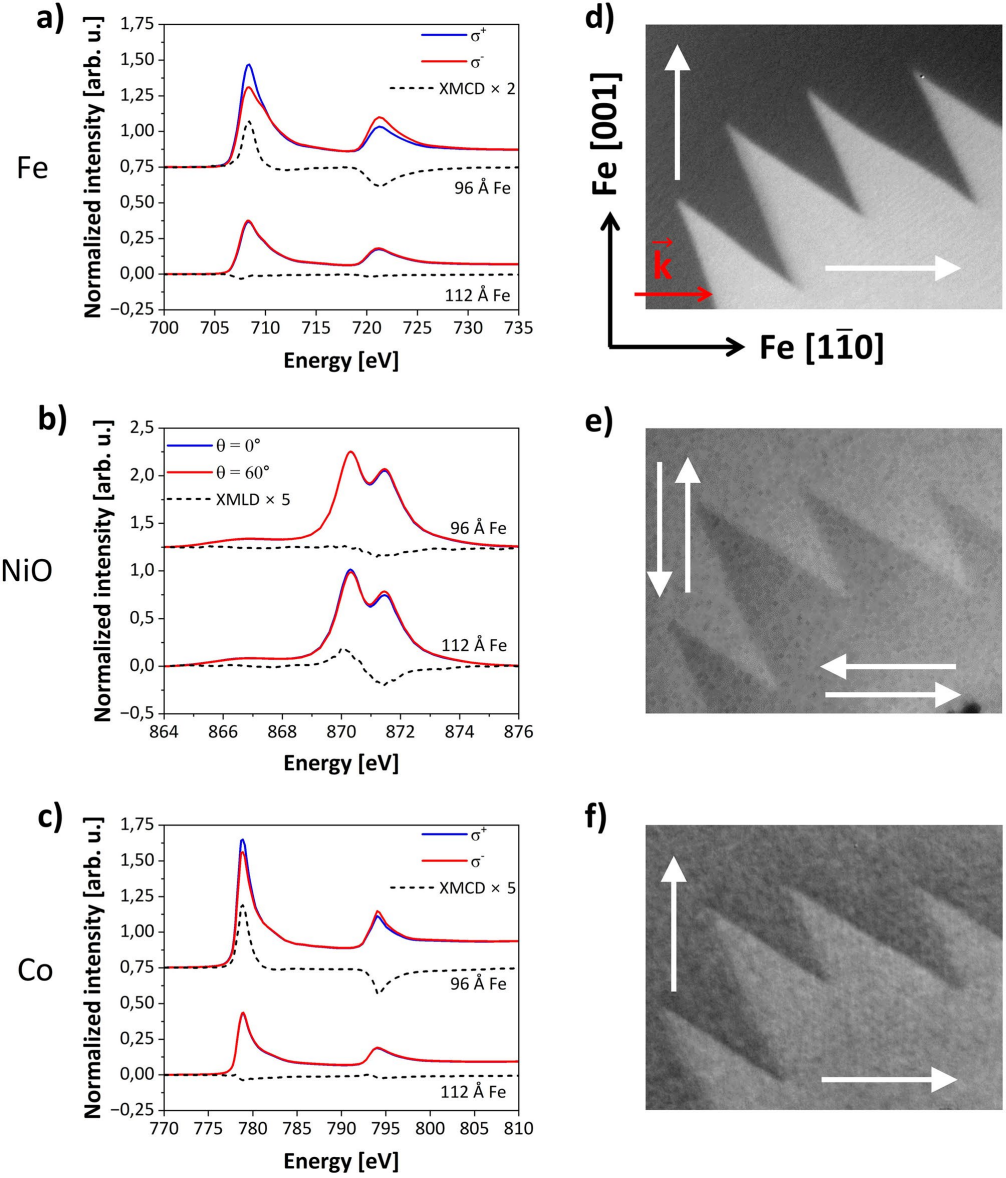
left column: XA spectra collected at Fe L_2,3_ (**a**) and Co L_2,3_ (**c**) absorption edges for Co(10 Å)/NiO(40 Å)/Fe(96 Å) (upper) and Co(10 Å)/NiO(40 Å)/Fe (112 Å) (lower). Spectra were registered at 80 K for left- and right-handed circular polarization ($\sigma^{ - }$ and $\sigma^{ + }$, respectively). Spectra measured on two sample regions are offset for clarity. Black dashed curves represent calculated XMCD for respective pairs of circular polarization spectra. Values of XMCD are multiplied to improve visibility; (**b**) Ni L_2_ XA spectra collected at 80K for thin and thick Fe part of the sample and two grazing angles θ for x-ray linear polarization with **E** || Fe[$1\overline{1}0$] in-plane direction. Curves under the spectra present the calculated XMLD. Values were multiplied for clarity.; right column: Element specific XPEEM images taken at the boundary between the Fe thickness of 104 Å and 108 Å. Fe L_3_ and Co L_3_ XMCD-PEEM images (**d** and **f**, respectively), (**e**) Ni L_2_ XMLD-PEEM image acquired at the same boundary area with x-ray linear polarization vector aligned along Fe[$1\overline{1}0$] direction. Field of view on the presented XPEEM images is 8 × 10 µm^2^.

Figure 2a presents the XA spectra collected at 80 K for photon energies scanned across the Fe L_2,3_ edges with right- and left- handed circular polarizations (${\sigma }^{+}$ and ${\sigma }^{-}$, respectively) (see Fig. S2(a) in Supplemental Materials for details of measurements). The spectra, after subtraction of the background were normalized to the highest intensity value of the average of the two spectra. Prior to the XAS measurements, external magnetic field of 140 mT was applied along the Fe[${1}\overline{1}{0}$] in-plane direction, thus the following XA spectra were measured in the remanent magnetization state of ferromagnets. For the trilayer with Fe thickness of 96 Å we noted a strong polarization dependence of the spectra for the photon beam propagation direction along Fe[${1}\overline{1}{0}$] (Fig. 2a, upper spectra) and a clear XMCD signal (Fig. 2a, upper, dotted line). In contrast, for 112 Å-thick Fe there was no detectable XMCD signal for the same measurement geometry (Fig. 2a, lower, dotted line). This indicates that the magnetization **M** in thin Fe is aligned along the x-ray incidence vector (**k**) (Fe [${1}\overline{1}{0}$] direction) while for thicker Fe layers **M** is parallel to Fe[001] direction and perpendicular to **k**. Thickness-induced spin reorientation transition in Fe layer was confirmed by element-specific measurements of hysteresis loops showed in the last part of this paper (see below). Ni L_2_ spectra collected under normal (θ = 0°) and 60° (θ = 60°) incident angle of x-rays from the sample regions with Fe thickness of 96 Å and 112 Å are shown in Fig. 2b. The spectra were recorded for linear polarization of the x-ray beam with the projection of the electric field vector **E** parallel to the Fe [${1}\overline{1}{\text{0}}$] in-plane direction (see Fig. S1(b) in Supplemental Materials for details on measurement geometry). Similarly to the XA spectra collected for circular polarization the spectra were normalized to the highest intensity value of the average of the two spectra. The spectra reveal a twin peak feature which is typical for the NiO XAS. The L_2_ ratio (RL_2_), defined as an intensity ratio of the higher energy peak (871.4 eV) to the lower energy peak (870.3 eV) can be employed to probe the orientation of magnetic moments in NiO^45^. In our study for *d*_*Fe*_ = 96 Å we noted RL_2_ = 0.8 while RL_2_ = 0.73 was registered for Fe thickness of 112 Å. As it was shown previously^34,35^, such a difference in RL_2_ is provoked by a change of orientation of NiO spins from Fe[${1}\overline{1}{\text{0}}$] (NiO[$\overline{2}{\text{11}}$]) to Fe[001] (NiO[${01}\overline{1}$]) direction due to the exchange coupling between FM and AFM layers. At first glance one would expect stronger dependence of L_2_ ratio on θ angle for NiO film grown on thinner Fe layer. However, we observed opposite behaviour, i.e. the XA spectrum collected for NiO grown on *d*_*Fe*_ = 112 Å is much more sensitive to the change of incident angle than the spectrum recorded for NiO/Fe(*d*_*Fe*_ = 96 Å). This result is a consequence of the fact that the XMLD asymmetry in NiO depends not only on the relative orientation of electric field **E** and AFM spins, but also the angular dependence with respect to the crystallographic axes. This conclusion is consistent with previous reports^35,46^. As we did not note polarization dependence of the spectra collected at Ni L_2_ edge for two opposite circular helicities (Fig. S2, Supplemental Materials), we do not expect ferromagnetic Ni in the sample. To investigate if changes in spin directions in NiO and Fe affect the orientation of magnetic moments within the top Co layer in Co/NiO/Fe we measured XA spectra on L_2_,_3_ absorption edges of Co (Fig. 2c). Similarly, to the Fe spectra, we noted a noticeable XMCD signal on the part of the sample with thinner Fe (Fig. 2c, upper), whereas no XMCD was detected on the sample region with thicker Fe (Fig. 2c, lower).

Transfer of magnetic properties from Fe film through NiO to Co top-most layer is also reflected in the domain structure of sublayers. The right column in Fig. 2 shows room temperature XMCD-PEEM (Fig. 2d and f)) and XMLD-PEEM (Fig. 2e) images collected at the boundary between Fe thickness of 104 Å and 108 Å, with a **k** photon beam direction parallel to the Fe[${1}\overline{1}{\text{0}}$] axis. The XMCD and XMLD contrasts were obtained as a result of the digital image processing described in Supplemental Materials. The characteristic zig-zag pattern indicates a 90° in-plane magnetization rotation^47–49^ at the boundary between *d*_*Fe*_ = 104 Å and *d*_*Fe*_ = 108 Å regions, which indicates that the critical thickness (*d*_*crit*_*)* of Fe at which spin reorientation transition occurs is 104 Å < *d*_*crit*_ < 108 Å (see Fig. S3 in Supplemental Materials). The antiferromagnetic domain structure of NiO was imaged using the XMLD-PEEM at the Ni L_2_ edge. The linear polarization of the incident x-ray beam was oriented in the plane of the sample along the Fe[${1}\overline{1}{\text{0}}$] (NiO[$\overline{2}{\text{11}}$]) direction. The domain pattern of underlaying Fe layer is directly reflected in the antiferromagnetic domains of the proximate NiO (Fig. 2e). Moreover, we noted the same zig-zag pattern for the XMCD-PEEM picture collected at the Co L_3_ edge, which confirms the imprinting of the Fe domain structure through the whole stack.

**Figure 3:**
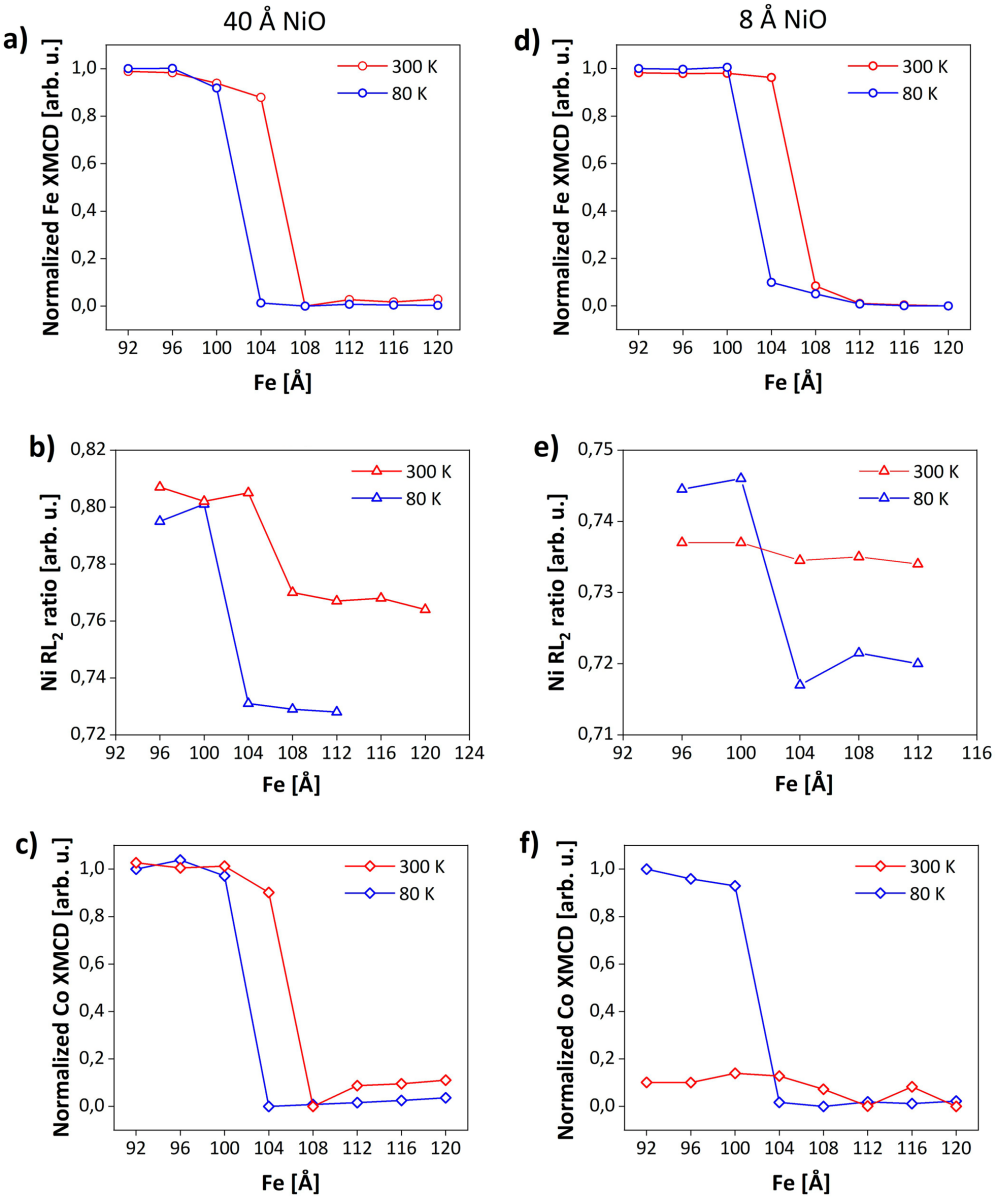
Fe thickness dependence of normalized XMCD determined from XAS measurements at the Fe L_3_ (**a**, **d**) and Co L_3_ (**c**, **f**) absorption edges at 80 K (blue) and 300 K (red); (**b**, **e**) The dependence of NiO RL_2_ as a function of Fe thickness at 80 K (blue) and 300 K (red). Left and right column shows results obtained for Co/NiO(40 Å)/Fe and Co/NiO(8 Å)/Fe, respectively.

Systematic XAS measurements of Co/NiO/Fe performed as a function of *d*_*Fe*_ revealed that the critical thickness of Fe defines not only the change of spin orientation in the Fe layer but also in NiO and Co components. Figure 3a and c show the evolution of XMCD asymmetry determined from the XAS intensity for two circular polarizations measured on Fe L_3_ (Fig. 3a) and Co L_3_ (Fig. 3c) absorption edges as a function of *d*_*Fe*_. The signals were normalized to the XMCD value obtained at 80 K for *d*_*Fe*_ = 92 Å. Since the measurements were performed along the Fe[${1}\overline{1}{\text{0}}$], an asymmetry value of 1 describes the orientation of magnetic moments parallel to the [${1}\overline{1}{\text{0}}$] direction of Fe while 0 value defines the perpendicular direction of magnetization (**M**||Fe[001]). Figure 3b shows the XMLD Ni L_2_ ratio determined from the XA spectra measured on the Fe stripes with different thicknesses. RL_2_(*d*_*Fe*_) dependencies were obtained from the spectra after subtraction of the background. During the measurement the incident beam was perpendicular to the (110) surface of the sample with linear polarization vector parallel to the Fe[${1}\overline{1}{\text{0}}$] direction, similar to the XAS measurements shown in Fig. 2b. The character of XMCD(*d*_*Fe*_) dependency noted for Fe (Fig. 3a) was followed by XMLD(*d*_*Fe*_) for Ni (Fig. 3b) and XMCD(*d*_*Fe*_) for Co (Fig. 3c). At 80 K, the magnetization of the Fe layer is parallel to [${1}\overline{1}{\text{0}}$] direction up to *d*_*Fe*_ = 100 Å, above which SRT to [001] direction occurs. An abrupt change in XAS signal observed in XMCD of Fe for 100 Å < *d*_*Fe*_ < 104 Å is replicated in RL_2_ dependence, i.e. a magnitude of Ni L_2_ ratio drops from 0.8 to 0.73 together with the increase in Fe thickness from 100 Å to 104 Å. This confirms that SRT in Fe is accompanied by the rotation of antiferromagnetic spins of NiO. An analogous trend noted for XMCD(*d*_*Fe*_) dependence for Co absorption edge (Fig. 3c) suggests that exchange coupling at the top AFM/FM interface results in simultaneous rotation of magnetic moments of Co film. A possible explanation of the change in the direction of **M** in Co in response to magnetization reversal in Fe could be the existence of “orange peel” effect^50^ in our sample. A way to prove that the change of XMCD signal of Co is a consequence of exchange coupling at the interfaces and is not related to the magnetostatic coupling is to heat the sample above T_N_. For T > T_N_, antiferromagnetic order vanishes, thus if the effect is related to the exchange coupling at the interfaces, XMCD of Co should not be sensitive to the SRT in Fe. For the trilayer with a NiO thickness of 40 Å the relatively high T_N_ > 400 K cannot be reached without intermixing in the multilayer structure or a reduction of the NiO layer. However, we proved that changes in magnetic properties of Co are induced by interfacial exchange coupling for the specially prepared sample, with thinner NiO sublayer, for which magnetic size effects reduce Néel temperature to below 300 K. For such Co/NiO(8 Å)/Fe sample, at 80 K, similarly to the results obtained for 40 Å of NiO we note that RL_2_(*d*_*Fe*_) dependence (Fig. 3e, blue) follows the changes in XMCD of Fe (Fig. 3d, blue). As the NiO layer is magnetic at low temperatures, the rotation of spins in Fe and NiO is accompanied by a variation of spins direction in Co (Fig. 3f, blue). At room temperature, RL_2_ is constant and remains insensitive to SRT in Fe, proving that the NiO layer is in a paramagnetic state. In this case, no change of XMCD(*d*_*Fe*_) in Co is observed (Fig. 3f, red). Thus, the changes in XMCD of Co are correlated with the magnetic properties of the NiO layer and do not originate from magnetostatic interactions between ferromagnets. As the strength of “orange-peel” coupling decays with an increase in interlayer thickness^50^, contribution from magnetostatic coupling in Co/NiO(40 Å)/Fe sample can be excluded.

At temperature of 300 K (Fig. 3a and d, red) we noted a shift in *d*_*crit*_ towards thicker Fe films in comparison with measurements performed at 80 K (Fig. 3a and d, blue). In the Co/NiO(40 Å)/Fe for *d*_*Fe*_ = 104 Å magnetic moments of Fe, NiO and Co are parallel to Fe[${1}\overline{1}{\text{0}}$] direction in contrast to a temperature of 80 K in which spins of FM and AFM layers were aligned along Fe [001]. An enhancement of the critical thickness at the elevated temperature is a consequence of the increase in [${1}\overline{1}{\text{0}}$] in-plane MA of Fe(110)^51^.

**Figure 4:**
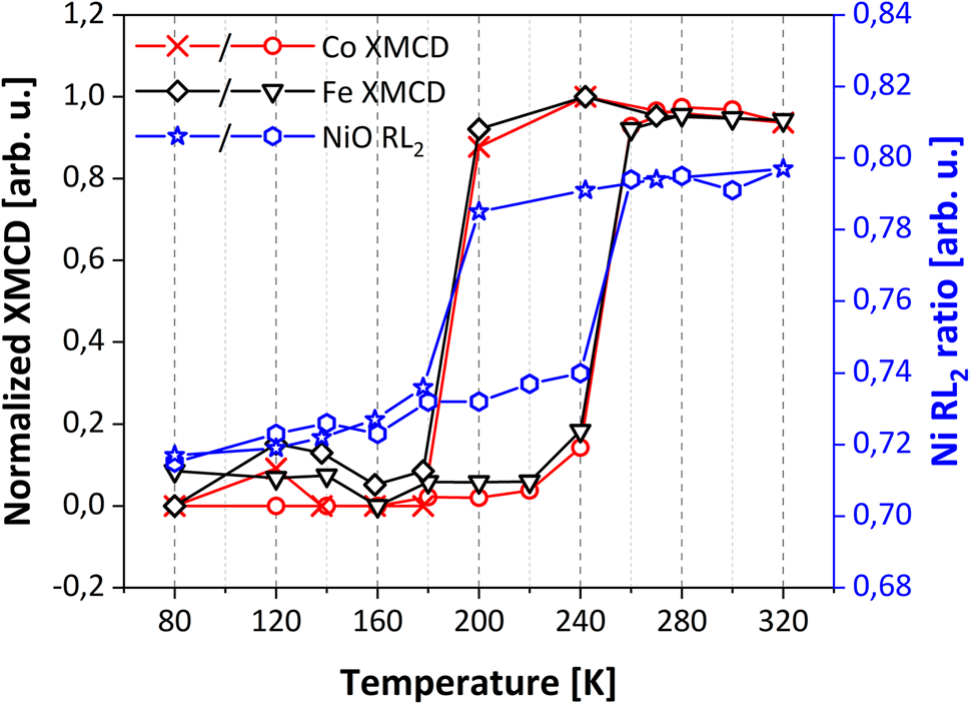
Temperature dependence of XMCD (black and red for Fe and Co respectively) and Ni L_2_ ratio (blue) in Co(10 Å)/NiO(40 Å)/Fe(104 Å) trilayer. The temperature-controlled bistability of the magnetic state is revealed by thermal hysteresis for all layers in the stack.

As surface and volume contributions to MA of Fe follow different temperature dependencies^51^, for Fe layers with thicknesses close to the *d*_*crit*_*,* which are characterized by a small MA, it is possible to induce SRT also by changing the temperature. As it was predicted theoretically^52^ and confirmed in experiments^34,53^ the temperature-induced SRT reveals hysteresis. Thus, one of two possible, orthogonal ferromagnetic states (with **M**||[${1}\overline{1}{\text{0}}$] and **M**||[001]) can be stabilized over a range of temperature. Recently we demonstrated that thermal hysteresis of the SRT in Fe is reproduced in the neighboring NiO layer in NiO/Fe/W(110)^34^. Consequently, depending on the history of the sample, it is possible to stabilize one of two orthogonal states in NiO at a given temperature. In the current study we show that due to the strong exchange coupling at the top Co/NiO interface, temperature-assisted hysteresis in Fe is transferred not only to the proximate NiO layer but to the Co cover layer as well. Figure 4 shows the temperature dependence of XMCD and L_2_ ratio determined for Co/NiO/Fe heterostructure for *d*_*Fe*_ = 104 Å. Temperature evolution of XMCD and RL_2_ was obtained from XAS measurements performed during cooling-heating thermal cycling. Within the temperature range (200–240) K it is possible to stabilize two orthogonal states of **M** in Fe layer, i.e. **M**_**Fe**_||Fe[${1}\overline{1}{\text{0}}$] persist down to 200 K during cooling cycle (Fig. 4., black squares) while **M**_**Fe**_||Fe[001] up to 240 K for heating (Fig. 4, black triangles).

Similarly to results obtained for NiO/Fe^35^, we noted that thermal hysteresis of the SRT in Fe is mimicked by the neighboring NiO layer. A pronounced change in RL_2_ is visible at T = 180 K and T = 260 K during cooling (Fig. 4, blue stars) and heating branches (Fig. 4, blue hexagons) which proves coupling between Fe and NiO sublayers. Moreover, for Co we noticed a similar hysteresis of the XMCD signal, e.g. a sudden change in XMCD signal was observed at the same temperatures at which SRT occurred in both Fe and NiO layers (Fig. 4, red). This indicates that the exchange coupling at FM/AFM and AFM/FM interfaces enables the possibility to stabilize two orthogonal magnetization states in all three sublayers within the (200–240) K temperature range, driven only by a change of the temperature.

The results of XAS measurements performed as a function of Fe thickness and for a given *d*_*Fe*_ as a function of temperature revealed that 90-degree SRT in Fe is transferred through the NiO layer to the top Co. In addition, element-specific XMCD and XMLD measurements collected as a function of the external magnetic field demonstrate that not only the direction of **M** but the strength of the anisotropy field is transferred to the top Colayer.

**Figure 5:**
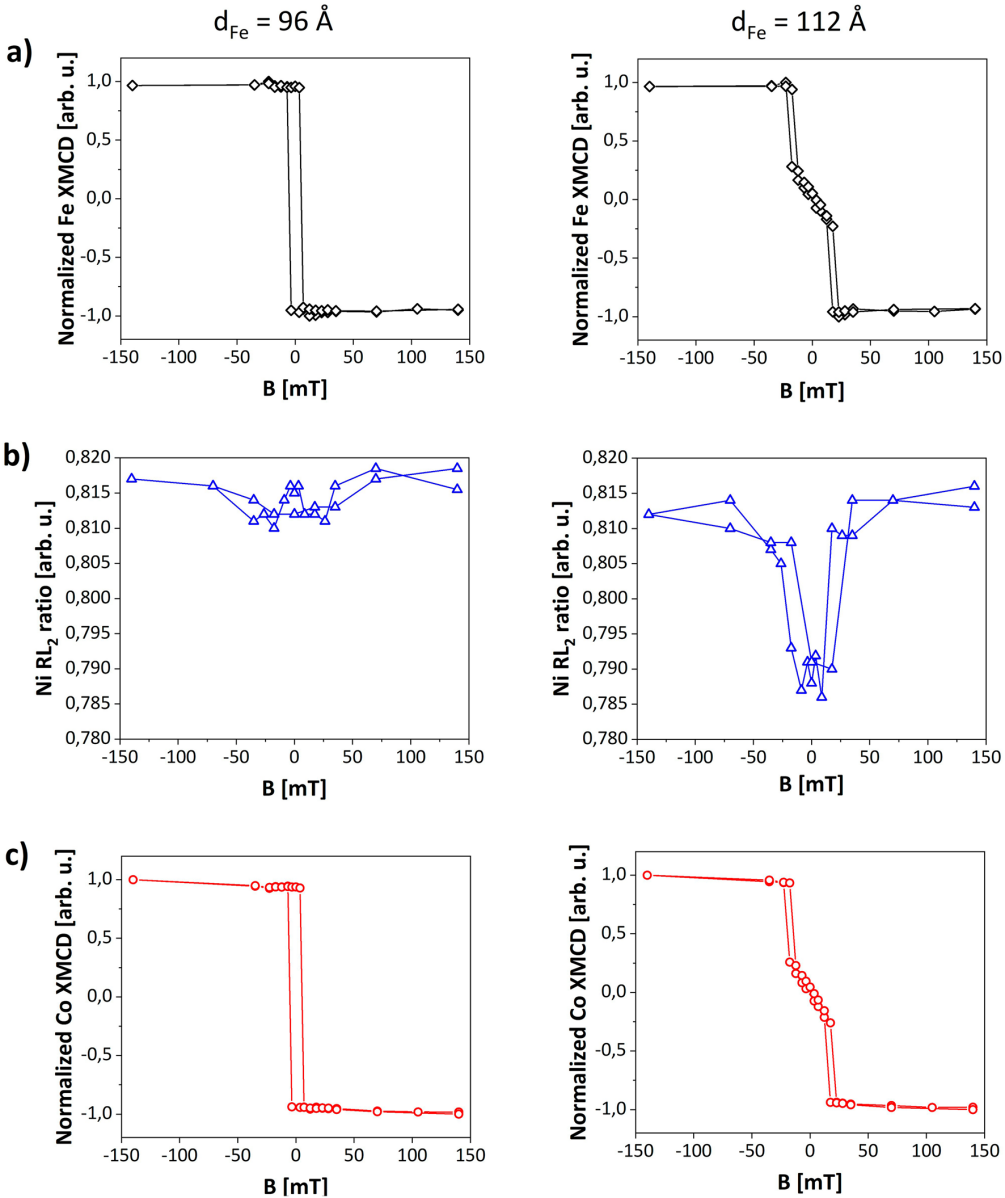
Element-specific XMCD (**a**, **c**) and XMLD (expressed by L_2_ ratio) (**b**) magnetic hysteresis loops of Fe, Co and NiO, respectively. Left and right column represents measurements performed for Co/NiO/Fe(96 Å) and Co/NiO/Fe(112 Å), respectively. During measurements an external magnetic field was applied parallel to Fe[$1\overline{1}0$] direction.

Figure 5 presents the element-sensitive magnetic hysteresis loops measured utilizing the XMCD for ferromagnetic sublayers and XMLD for the antiferromagnetic NiO interlayer. XMCD measurements were carried out in grazing geometry with the x-ray beam illuminating the sample at $\theta =60^\circ$, where $\theta$ is the angle between the incident beam and the sample surface normal (as shown on Fig. S1(a), Supplemental Materials). During the measurements the in-plane component of the external magnetic field was applied along Fe[${1}\overline{1}{\text{0}}$] direction. Note that although a non-zero out-of-plane component of magnetic field exists for such measurement geometry, a significantly higher magnitudes of external fields are necessary to rotate the magnetic moments of Fe out of the (110) sample plane. Thus, the out-of-plane component of the magnetic field has no effect on Fe magnetization and can be omitted. On the part of the sample with *d*_*Fe*_ = 96 Å (Fig. 5a, left), we obtained a square hysteresis loop at Fe L_3_ absorption edge, while for Fe thickness of 112 Å a typical hard axis loop with almost zero magnetization in remanence state and anisotropy field of 10 mT was registered (Fig. 5a, right). This confirms that magnetization of Fe layer with a thickness of 96 Å is aligned along Fe [${1}\overline{1}{\text{0}}$] whereas **M**_**Fe**_ of the layer with *d*_*Fe*_ = 112 Å is parallel to Fe[001] direction. As the XMLD is insensitive to the 180° reversal of magnetic moments we do not observe a significant dependence of XMLD signal on magnetic field (Fig. 5b, left) for Co/NiO/Fe(96 Å). On the contrary, for Co/NiO/Fe(112 Å) an in-plane 90-degree switching of NiO spins is visible in the magnetic field evolution of XMLD signal (Fig. 5b, right). This can be understood if we consider that AFM spins follow the reversal of magnetization of the Fe layer for which the magnetic field drags **M**_**Fe**_ towards the hard axis direction^35^. Please note, that independently on the Fe thickness the measured hysteresis loops are fully symmetric with respect to zero-field axis, i.e. they do not exhibit exchange bias field. This means that the antiferromagnetic NiO spins are rotatable and follow any reorientation of the Fe magnetization, in contrast to the frozen AFM spins^11^ and large exchange bias in recently reported isostructural CoO(111)/Fe(110) system^54^. The shape of normalized hysteresis loops registered at the Co L_3_ edge (Fig. 5c) is identical to the loops collected at Fe L_3_ edge. In particular, for the hard axis loops we noted the same saturation field for Co and Fe layers (compare black and red loop in the right column in Fig. 5). This shows that not only the direction of MA but also anisotropy field strength is transferred from Fe layer through NiO to Co film. Magnetic proximity effect in Co/NiO/Fe trilayer is responsible for creation of the artificial two-fold magnetic anisotropy in hexagonal cobalt film with six-fold crystalline symmetry.

## Conclusions

In summary, we showed that exchange coupling at NiO/Fe and Co/NiO interfaces along with well-defined and controllable magnetic anisotropy of Fe layer determines the magnetic properties of both NiO and Co layers in Co/NiO/Fe trilayer structure. XMCD and XMLD measurements performed for Co/NiO/Fe epitaxially grown on W(110) showed that thickness-driven spin reorientation transition in Fe is transferred not only to the NiO layer but also to ferromagnetic Co layer as well. We proved that magnetic anisotropy of Co overlayer can be precisely controlled by tuning the properties of bottom Fe layer. Specifically, reorientation processes in Co can be triggered by changing the Fe thickness or by variation of the temperature. The measurements of element-specific magnetic hysteresis loops revealed that besides MA, magnetic anisotropy field is transferred from Fe to Co layer. This result shows that Co film can be treated as a probe layer of magnetic properties of buried NiO and Fe layers which is valuable for surface-sensitive techniques, e.g. spin-polarized microscopy (STM) or spin-polarized low electron energy microscopy (SPLEEM). Use of conducting Co probe layer that mimics the magnetic state of the buried films enables to visualize the spin structure in buried NiO layers with laboratory methods. Moreover, our results are important for the process of imprinting of spin structure between neighboring magnetic systems with different type of magnetic order. In particular, in the case of AFM-FM systems, previous studies concerning continuous films have reported the imprinting of AFM domain structure onto the FM component^55–57^. Our results demonstrate a step further as the spin structure of the bottom Fe layer is not only transferred across the AFM NiO layer to the top Co overlayer but also the response of spins of all magnetic components of the system to the external magnetic field is unified. Such result can be exploited in grafting of more complex spin textures such as vortices^11^ or skyrmions from a ferromagnet to antiferromagnet and further to another ferromagnet. Finally, we showed that such strong interaction between ferromagnetic layers, mediated by antiferromagnetic spacer, can be turned on or off by antiferromagnetic size effects.

### Supplementary Information

Supplementary Information.

## Data Availability

The data that support the findings of this study are available from the corresponding author upon reasonable request.
