# Supplementary material for: Transfer of magnetic anisotropy in epitaxial Co/NiO/Fe trilayers
Source: Sci Rep. 2024 Jan 19;14:1680. doi: 10.1038/s41598-024-51896-w (PMC10798992; doi:10.1038/s41598-024-51896-w)
Supplement: Supplementary file 1 — Supplementary Information. [file 41598_2024_51896_MOESM1_ESM.pdf]

Transfer of magnetic anisotropy in epitaxial Co/NiO/Fe trilayers

M. Szpytma [1)*], M. Ślęzak [1)], W. Janus [1)], H. Nayyef [1)], T. Ślęzak [1)], A. Mandziak [2)], M. Zając [2)], D. Wilgocka-Ślęzak [3)], T. O. Menteş [4)], M. Jugovac [4)], A. Locatelli [4)] and A. Kozioł-Rachwał [1)]

[1)] Faculty of Physics and Applied Computer Science, AGH University of Krakow, Kraków, Poland

[2)] National Synchrotron Radiation Centre SOLARIS, Jagiellonian University, Kraków, Poland

[3)] Jerzy Haber Institute of Catalysis and Surface Chemistry, Polish Academy of Sciences, Kraków, Poland

[4)] Elettra – Sincrotrone Trieste, Basovizza, Trieste, Italy

* corresponding author: mszpytma@agh.edu.pl

XMLD/XMCD – XPEEM asymmetry calculations

The XMLD-PEEM images were obtained by calculating the asymmetry of two images according to the following intensity I relation:

$$I_{XMLD} = \frac{I(h\nu_1) - I(h\nu_2)}{I(h\nu_1) + I(h\nu_2)} \,,$$

where $I(h\nu_1)$, $I(h\nu_2)$ correspond to the signal intensities of images taken at the energies $h\nu_1$ and $h\nu_2$ of absorption peaks within the Ni L_2 edge (867.9 eV and 869.1 eV, respectively).

XMCD-PEEM asymmetry images of Fe and Co were collated at L_3 Fe and Co absorption edges as the difference between right(σ^+) and left (σ^-) handed circular polarization images divided by their sum, at fixed photon energy, according to the formula:

$$I_{XMCD} = \frac{I(\sigma^+) - I(\sigma^-)}{I(\sigma^+) + I(\sigma^-)} \,.$$

XAS measurement geometries

XAS measurement geometry at PIRX beamline at SOLARIS National Radiation Centre is presented in Fig. 1S. Fig 1S(a) depicts the geometry of XMCD measurements, while Fig 1S(b) shows the geometry used for collecting the XMLD spectra of antiferromagnetic NiO. In the case of XMCD, spectra with right-handed (σ^+) and left-handed (σ^-) circular polarizations were collected at Fe $L_{2,3}$ and Co $L_{2,3}$ absorption edges. To ensure the sensitivity to spin alignment in the plane of the sample, incident X-rays beam was set at an angle of 60° with respect to the sample surface normal. During the measurements the **k** vector of incoming photons was parallel to the Fe $[1\bar{1}0]$ direction.

To probe the NiO spin structure, XAS spectra were collected using a linearly polarized x-ray beam with a photon energy corresponding to the Ni L_2 edge. XA spectra were collected for the incidence angle θ = 0° and θ = 60°, where θ is defined as the angle between the propagation direction of the x rays and the sample surface normal. During measurements performed for θ = 0° polarization vector **E** was parallel to the Fe [1$\bar{1}$0] (NiO [$\bar{2}$11]) in-plane direction. For θ = 60^o a noticeable out-of-plane component of the electric field vector appeared while the projection of the **E** on the NiO(111)||Fe(110) sample plane was parallel to the Fe [1$\bar{1}$0] (NiO [$\bar{2}$11]) direction. To illuminate the same sample area for spectra collected at θ = 0° and θ = 60° for measurements performed at θ = 60° the footprint of the beam was limited by the slits.

To verify if there are Ni ferromagnetic moments in the sample XA spectra at Ni L2 edge were collected for right- and left- handed circular polarizations (Fig. S2, blue and red). As we did not note polarization dependence of the spectra, we do not expect contribution from metallic Ni in the sample.

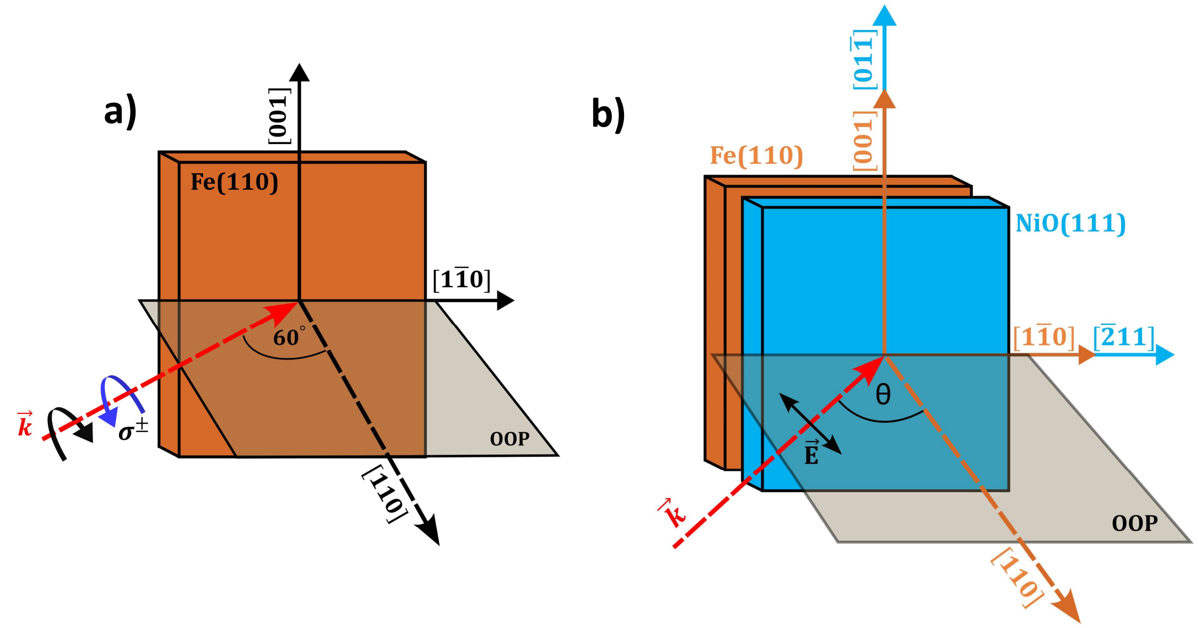

Figure S1 Schematic representations of XAS geometries intended for measurements of: a) XMCD, b) XMLD absorption spectra.

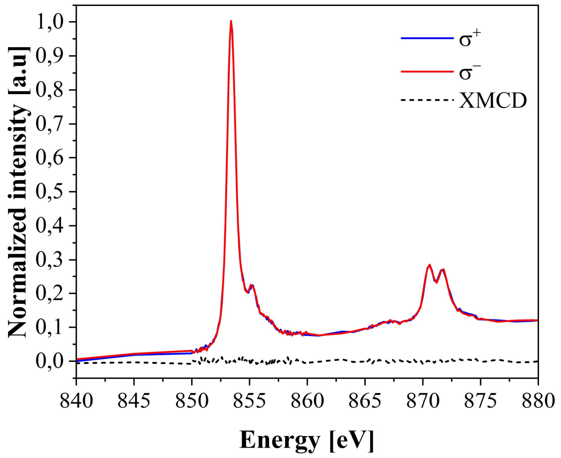

Figure S2 XAS spectra collected at Ni L_2 edge for two circular polarizations (red and blue).

Spin reorientation transition (SRT) in Fe

Figure S3 shows schematically the border between the d_{Fe} = 104 Å and d_{Fe} = 108 Å at which room-temperature XPEEM image was collected. On schematic drawing of the sample Fe thickness dependence of normalized XMCD measured at Fe L_3 was presented, which shows change of XMCD from 1 to 0 when the Fe thickness is increased from d_{Fe} = 104 Å to d_{Fe} = 108 Å. Element specific room-temperature XMCD hysteresis loops collected for mentioned Fe thicknesses recorded with an in-plane magnetic field applied along Fe[1$\bar{1}$0] change character from easy to hard together with an increase in Fe thickness, which proves existence of SRT in imaged area.

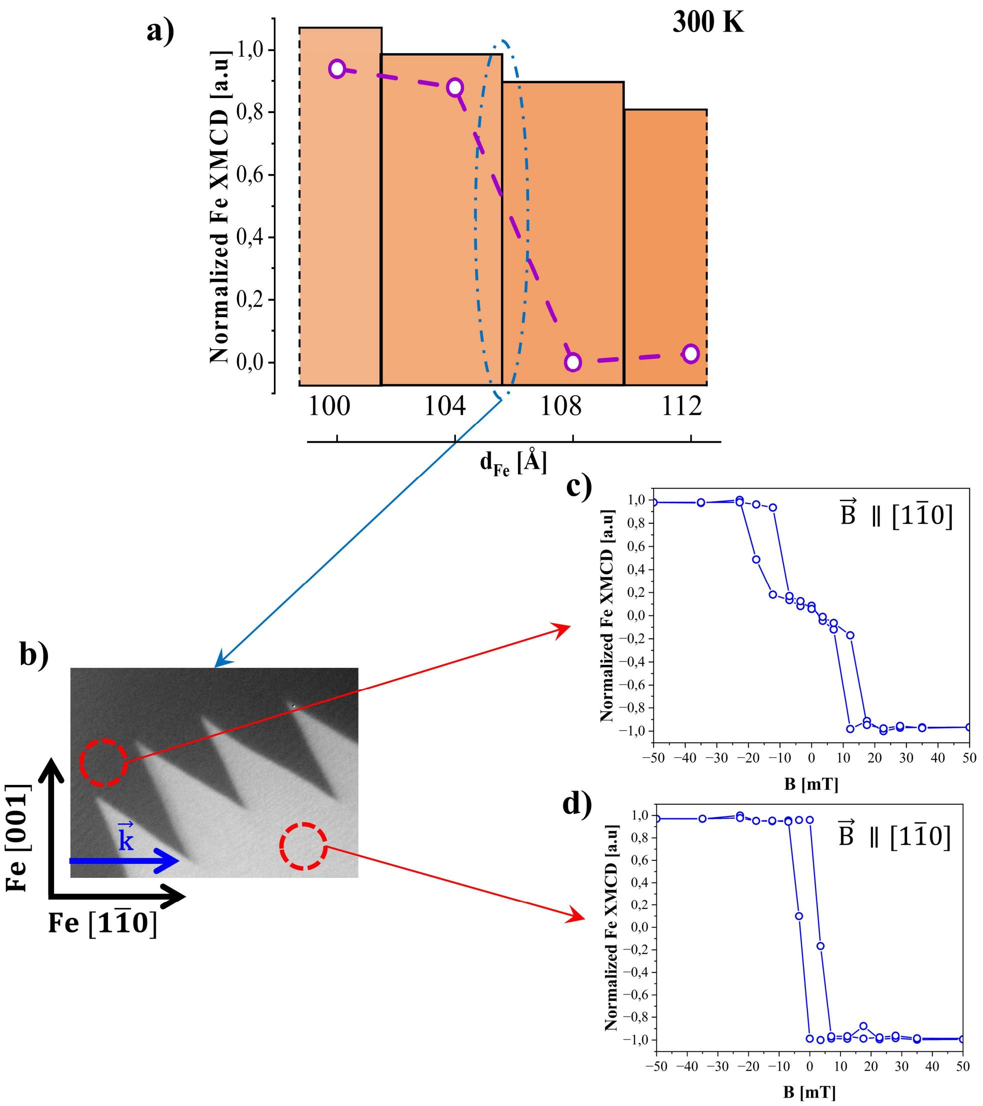

Figure S3 (a) Schematic drawing of the border between the d_{Fe} = 104 Å and d_{Fe} = 108 Å shown together with normalized XMCD measured at Fe L_3 (purple, dotted line), (b) room-temperature X-PEEM image collected at the border between the d_{Fe} = 104 Å and d_{Fe} = 108 Å, (c) and (d) Element-specific room-temperature XMCD hysteresis loops collected for d_{Fe} = 104 Å and d_{Fe} = 108 Å registered with an in-plane magnetic field applied along Fe[1$\bar{1}$0].

Influence of Au capping on magnetic properties of the Co/NiO/Fe

Figure S4 shows normalized XMCD dependencies at Fe L_3 and Co L_3 absorption edges and dependence of NiO RL_2 as a function of d_{Fe} at 300 K before and after deposition of top Au layer. Additionally, XMCD dependencies were obtained for low temperature measurements (80 K). We did not note any changes in XMCD and XMLD dependencies after capping the Co/NiO/Fe heterostructure with Au layer. Thus, capping layer do not influence magnetic properties of the stack.

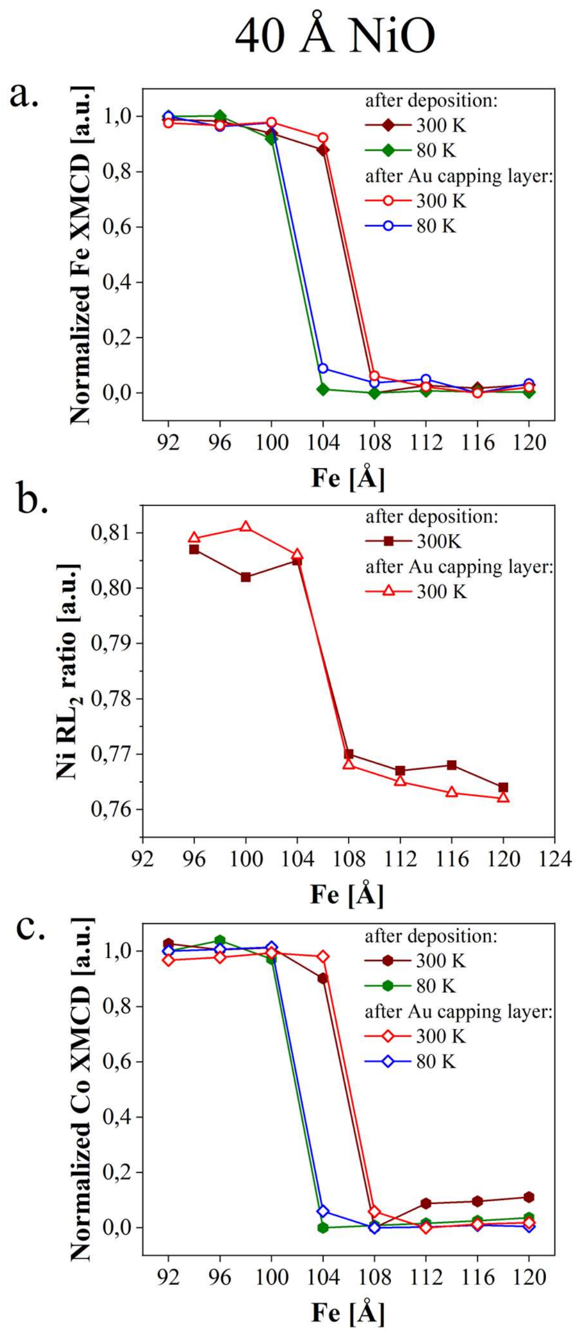

Figure S4 Fe thickness dependence of normalized XMCD determined from XAS measurements at the Fe L_3 (a) and Co L_3 (c) absorption edges at 80 K (blue and green) and 300 K (red and dark red) before (dark red, green) and after (red, blue) capping the Co/NiO/Fe heterostructure with 1 nm of Au. (b) The dependence of NiO RL_2 as a function of Fe thickness at 300 K before (dark red) and after (red) capping the heterostructure with 1 nm of Au.
